# For the sake of longevity: eating less and eating at the right time

**DOI:** 10.1038/s41392-022-01163-z

**Published:** 2022-09-09

**Authors:** Fei Sun, Si Wang, Guang-Hui Liu

**Affiliations:** 1 Beijing Institute for Stem Cell and Regenerative Medicine, Beijing, 100101 China; 2 Department of Cell Biology, Duke University Medical Center, Durham, NC USA; 3 Advanced Innovation Center for Human Brain Protection, and National Clinical Research Center for Geriatric Disorders, Xuanwu Hospital Capital Medical University, Beijing, 100053 China; 4 Aging Translational Medicine Center, International Center for Aging and Cancer, Beijing Municipal Geriatric Medical Research Center, Xuanwu Hospital, Capital Medical University, Beijing, 100053 China; 5 The Fifth People’s Hospital of Chongqing, Chongqing, 400062 China; 6 State Key Laboratory of Membrane Biology, Institute of Zoology, Chinese Academy of Sciences, Beijing, 100101 China; 7 University of Chinese Academy of Sciences, Beijing, 100049 China

**Keywords:** Senescence, Diseases

In a recent issue of *Science*, Acosta-Rodriguez et al. defined a feeding paradigm to maximize the effects of caloric restriction (CR) on lifespans. Aligning feeding with the circadian rhythm and imposing >12-hour fasting daily on caloric-restricted animals can optimally extend lifespans.

While embracing the projection of our life expectancy in the last 100~150 years, we are facing aging, an all-new challenge in human evolution. Aging-associated diseases have gradually replaced communicable diseases, becoming the top cause of death globally. The increasing social and medical burdens call for not only novel therapies for chronic diseases but also early interventions that mitigate the progression of aging and extend life and health spans.

Studies involving a wide range of species have illuminated CR as one of the feasible strategies to promote longevity in animals.^1^ Animals subjected to CR were demonstrated with ameliorated aging-associated hallmarks, improved metabolism, and better immune functions. Although restricting daily energy intake is the primary goal, classical CR protocols inevitably increase fasting periods and limit food-accessible time. Each of these factors was known to impact body weight and animal health significantly. Thus, disentangling the individual contribution of these parameters within classical CR protocols will facilitate the delineation of mechanisms underlying their beneficial impacts on lifespans. Further exploring the combined effects of different feeding schemes will guide the design of an optimal CR feeding paradigm for extending longevity.

**Fig. 1:**
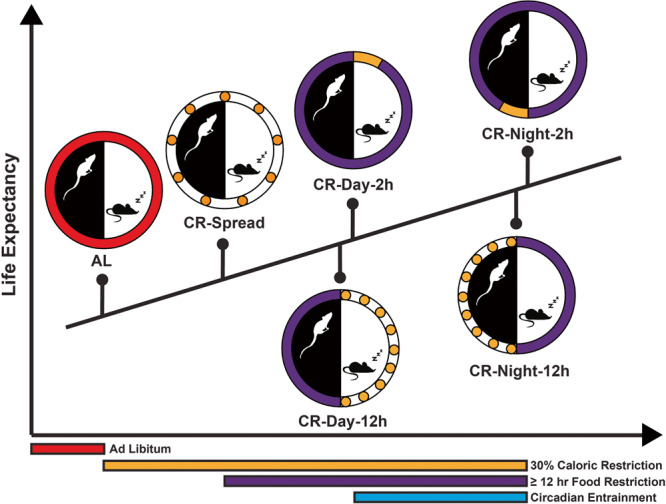
Impacts of diet regimens on life expectancy. Five different CR feeding paradigms were designed to assess their beneficial effect on longevity. Male C57BL/6J mice were subjected to the AL diet for the first six weeks and then switched to a 30% CR purified diet for the rest of their life. Compared with ad libitum feeding, controlling daily calorie intake solely can improve the median lifespans by about 10%. Feeding CR diet to animals restrictedly in a 2-hour or 12-hour period during the day and minimally fasting animals for 12 h daily further extend median lifespans by another 10%. However, the most significant promotion of longevity was observed when combining CR with > 12-hour fasting interval and nocturnal feeding. Entraining circadian rhythm with the nocturnal feeding and diurnal fasting cycle can ultimately increase lifespans by about 35% in animals. AL Ad libitum, CR Caloric restriction

To dissect the impact of calorie intake, fasting length, and feeding time on lifespans, Acosta-Rodriguez et al. applied five CR feeding schemes on male C57BL/6J mice with the ad libitum (AL) feeding animals as the control.^2^ While the total calorie intake is the same for each CR group, the duration of fasting and time of feeding are different (Fig. 1). The CR-spread group was fed every 160 min throughout the day to abrogate the rhythm in food intake and prevent fasting. Two CR groups were fed every 90 min for 12 h during the daytime (CR-Day-12h) or the nighttime (CR-Night-12h) to impose a 12-hour fasting period on mice. The last two groups were fed only once at the beginning of the day (CR-Day-2h) or the night (CR-Night-2h). Since mice under CR treatment consume all food within 2 h after feeding, they were self-imposed to more than 22-hour fasting daily. Including diurnal and nocturnal feeding regimens in this study further provides a unique opportunity to assess the significance of feeding rhythm on longevity.

Following mice for almost four years, the authors discovered a wide range of lifespan extensions in CR groups (Fig. 1). Consistent with previous studies, restricting calorie intake solely is sufficient to extend longevity. However, when combining CR with fasting and nocturnal feeding, lifespan changes more drastically. Although >12-hour fasting between meals without CR exerts diverse effects on animal lifespans, fasting mice for 12 h and 22 h daily equally boost lifespans in this study, suggesting that 12-hour fasting is long enough for prolonging lifespans under the CR protocol. Further comparing the diurnal with the nocturnal CR paradigm, the authors observed a significant increase in the median lifespans from ~950 to ~1060 days. These findings indicate that CR and time-restricted feeding act synergistically to extend lifespans, and a CR program integrating circadian entrainments is the optimum for delaying aging.

To mechanistically address how CR rescues aging in animals, the authors assessed metabolic markers in young and old mice plasma. As expected, the long-living CR mice maintain high sensitivities to insulin and glucagon-like peptide-1 to mediate glucose homeostasis. Analyzation of liver transcriptomes in young and old mice revealed that 50% of the age-associated genes identified in the AL group restore their expression in every CR group. Further monitoring the transcriptomic changes in the liver every 4 h for 48 h, the authors identified hundreds of cycling genes. With the progression of aging, the amplitude of the circadian rhythmicity of these genes significantly declines. More intriguingly, daytime feeding induced opposite or divergent circadian expression of some cycling genes, whereas night feeding robustly boosts the circadian amplitudes in the old mice. Although the authors did not explicitly explain how feeding paradigms alter mice’s behavior and overall well-being during aging, they did demonstrate that synchronizing the low-energy fasting protocol with circadian rhythm effectively sustains the molecular signatures of the young animals to the old.

In conclusion, this study introduced a new feeding paradigm for exploring the collective effects of energy restriction, time-restricted feeding, and circadian intervention on a broad range of animal health. In agreement with a recent clinical trial^3^, time restriction does not add to the beneficial effect of CR on body weight. However, animal lifespans are extended when CR feeding schemes align with the circadian clock. This implies that the same feeding paradigm may diversely impact different health parameters. We suspect that the effect of feeding regimes on tumor onsets may determine animal lifespans, given that liver cancer is the primary cause of death in Acosta-Rodriguez’s study. As aging stimulates tissue or cell type-specific changes in animals^4^, different feeding paradigms likely mitigate aging in a tissue or cell type-specific manner. Evaluating the health benefits separately in each organ and considering the effects collectively on animal well-being is critical when selecting feeding paradigms for therapeutic interventions.

This study provided limited clues on how dietary restrictions improve animal lifespans mechanistically. Feeding paradigms can alter circadian oscillation in peripheral organs like the liver in mice and eyes in drosophila. Disrupting peripheral circadian rhythm in some cases is sufficient to reduce longevity. However, it remains unclear how feeding interacts with the circadian rhythm in peripheral organs and how elevated circadian rhythm benefits longevity. A recent study indicates that the dysregulation of core clock protein destabilizes heterochromatin structures and induces cellular senescence in human mesenchymal stem cells in vitro. Exogenously boosting CLOCK expression can enhance cartilage regeneration in aged mice.^5^ Therefore, analyzing the epigenetic changes in aging mice after different feeding paradigms may provide invaluable insights to elucidate the anti-aging mechanism underlying circadian interventions.
